# Benign Paroxysmal Positional Vertigo (BPPV) After Concussion in Two Adolescent Players During a Rugby Game

**DOI:** 10.7759/cureus.33402

**Published:** 2023-01-05

**Authors:** Khalid Bashir, Abdulla Yousuf, Hany A Zaki, Amr Elmoheen

**Affiliations:** 1 College of Medicine, Qatar University, Doha, QAT; 2 Department of Emergency Medicine, Hamad Medical Corporation, Doha, QAT; 3 Department of Medical Education and Simulation, Hamad General Hospital, Doha, QAT

**Keywords:** emergency department, trauma, rugby, epley maneuver, bppv

## Abstract

Benign paroxysmal positional vertigo (BPPV) is a medical condition where patients develop symptoms of vertigo, “room spinning,” associated with nausea and vomiting. BPPV is believed to be caused by a disturbance in the inner ear vestibular system. Trauma has been recognized as one of the risk factors for this condition. BPPV can be easily diagnosed and treated by bedside maneuvers. Due to a lack of awareness among some treating clinicians, patients may have to wait for a long time before the correct management is offered. We share two cases of BPPV in 15- and 16-year-old male school students who developed posterior canal BPPV following a head injury during a rugby game. Both patients continue to have vertigo symptoms for several weeks before the final diagnosis. BPPV symptoms completely resolved following the Epley maneuver. Frontline clinicians need to diagnose and treat BPPV early to prevent the persistence of these debilitating symptoms. As far as we are aware, no previous study has published the occurrence of BPPV in young adolescent rugby players.

## Introduction

Patients with dizziness are commonly seen in many medical specialties, such as family medicine, ear, nose, and throat (ENT), neurology, emergency medicine, physical therapy, and neurosurgery. Approximately 90 million patients annually are seen with dizziness in the United States alone [1]. The condition is more prevalent in those over 50 years. Benign paroxysmal positional vertigo (BPPV) is a subgroup of dizziness where the patient describes an illusion of movement associated with certain head positions, such as looking up, bending down, changing position in the bed, and head rotation. The exact cause is still unknown. However, it is strongly believed to be caused by free-floating calcium particles within the semicircular canal portion of the inner ear. BPPV can be easily diagnosed by taking an appropriate history and performing a bedside Dix-Hallpike maneuver. Posterior canal BPPV is the commonest subtype and can be treated by bedside canalith repositioning maneuvers (CRM). The Epley maneuver is one of the well-recognized CRM that helps move the crystals out of the semicircular canals back to the utricle. Due to a lack of training, some physicians find it difficult to treat BPPV by physical maneuvers; instead, they perform computed tomogram (CT) scans of the brain and prescribe medications that temporarily relieve the symptoms [2,3]. “Concussion is a condition in which there is a traumatically induced alteration in mental status, with or without an associated loss of consciousness (LOC)” [4]. BPPV frequently develops in the pediatric population following concussion, accounting for almost one-third of the cases. After headaches, BPPV is the second most common presenting complaint of concussion. Unfortunately, many children with BPPV are not diagnosed until many weeks after the injury [5]. Protective headgear, “rugby scrum caps” (RSC), is usually worn by players to protect against head injury. RSC has repeatedly been shown to protect against superficial head injuries [6]. It is important to create awareness among the clinicians who deal with pediatric head injury concussion so that BPPV can be diagnosed and treated early to prevent long-term disability. Most clinicians who provide regular treatment to patients with concussions have little or no experience managing BPPV, which may lead to delays in managing BPPV [2].

We report two cases of BPPV in 15- and 16-year-old male adolescent schoolchildren who developed a brief LOC after a head-on collision during rugby matches. One player developed several episodes of vomiting. Both players were wearing RSC. They were stopped from continuing the game by the team doctor. Both players were then transferred to the local emergency department (ED) for a professional opinion. Several weeks after the injury, both went back to the ED with severe vertigo after getting out of bed, where both underwent a Dix-Hallpike test, with a confirmed right-sided posterior canal BPPV diagnosis. They were treated with the Epley maneuver, which completely resolved symptoms ultimately.

## Case presentation

In case 1, a previously healthy 16-year-old adolescent playing rugby in the front row collided with a player from the opposite team. He was wearing RSC. He fell to the ground, followed by a brief loss of consciousness. He developed several episodes of vomiting. Examination by the team doctor revealed a minor head injury leading to a concussion. Due to the frequency of vomiting, he was transferred to the local emergency department (ED). He was assessed and underwent a head CT scan, which was reported as normal. He was kept in the hospital for 24 hours in the observation unit of the emergency department. After 24 hours, he was deemed fit to be discharged home. Eight days after being discharged, he developed severe room spinning vertigo after getting out of bed associated with vomiting. He was seen at home by the family physician, and oral medication (betahistine 8 mg TDS) for vertigo was given, which improved his vertigo symptoms. During the next few weeks, he experienced several brief episodes of vertigo. He again consulted the family physicians, who reassured him and recommended continuing with the prescribed medications. Five weeks after the injury, while getting out of bed, he developed the worst vertigo symptoms; he was taken to the ED by the emergency ambulance crew, where he was diagnosed with right-sided posterior canal BPPV, and the Epley maneuver was offered, which resulted in the complete resolution of symptoms. He was followed up six weeks later in the outpatient clinic of the ENT department, and there was no recurrence of symptoms.

In case 2, a previously healthy 15-year-old collided with a player from the opposite team. There was a brief loss of consciousness, but no vomiting. The team doctor diagnosed him with a minor head injury, and he was not allowed to continue the game due to loss of consciousness. He was seen at the local ED and was discharged home with a diagnosis of a minor head injury. He subsequently developed several episodes of vertigo at home but did not seek medical advice. Six weeks after the injury, he developed severe vertigo with room spinning and vomiting while getting out of bed in the morning. He was taken to the ED via ambulance, where he underwent a head CT scan, which was reported as unremarkable. He was subsequently diagnosed with right-sided posterior canal BPPV. He was treated with the Epley repositioning maneuver with the resolution of symptoms. He was discharged home with oral betahistine 8 mg TDS for eight weeks. There was no recurrence of symptoms at the eight-week follow-up in the ENT clinic.

## Discussion

**Figure 1:**
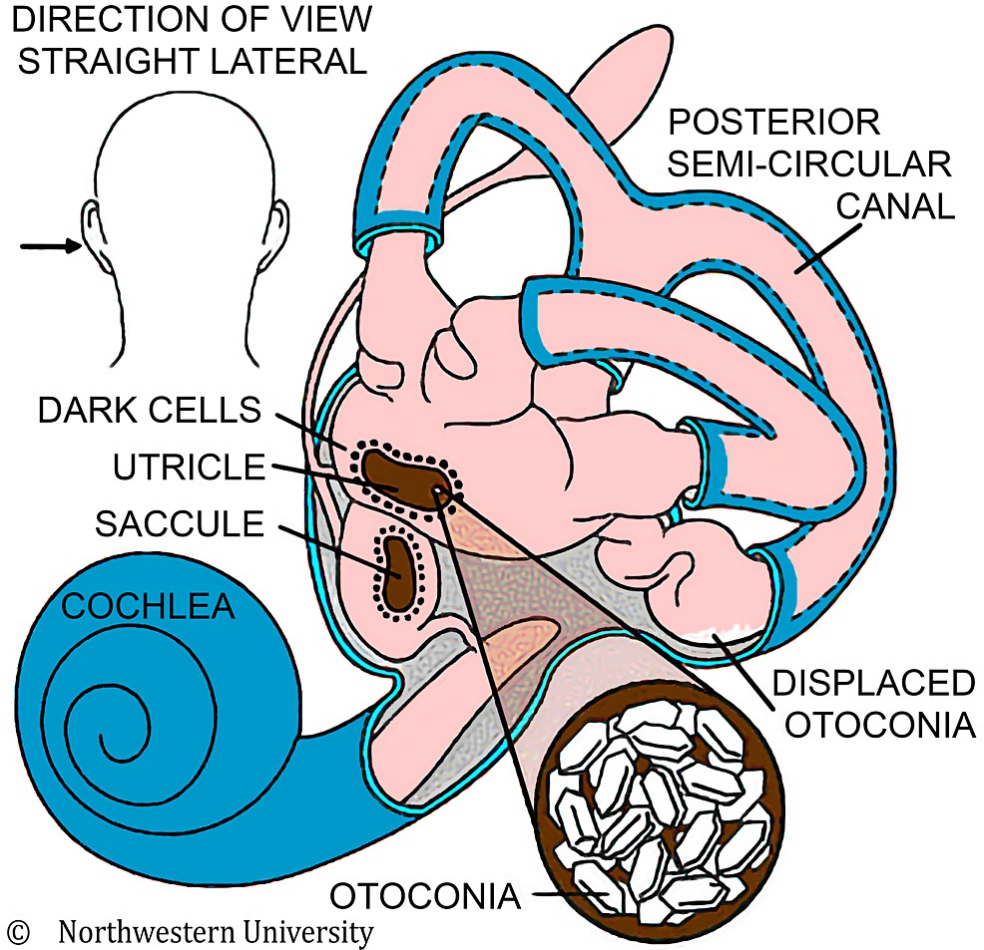
Anatomy of the left inner ear showing displaced otoconia from the utricle and saccule to the posterior semicircular canal Source: [7]

BPPV is extremely common in pediatric patients with concussions following head injury. Vertigo symptoms are induced by the movement of free-floating calcium crystals, “otoconia,” in the semicircular canals (Figure 1) [7].

Posterior canal BPPV is the most common subtype of BPPV and can be easily diagnosed with the Dix-Hallpike test. Most patients experience resolution of symptoms after the Epley maneuver, but minor symptoms may take several hours or even days to resolve. Occasionally, patients may require maneuvers for several days before complete recovery. Due to the lack of awareness of managing BPPV among treating clinicians, some patients take a long time to reach the final diagnosis. Increasing awareness among clinicians treating concussions is important, as early repositioning maneuvers can reduce morbidity.

In one study, almost 30% of pediatric patients developed BPPV following concussion [8]. Except for a few who recovered spontaneously, all patients were treated successfully with the beside repositioning maneuvers. In the same study, the authors concluded that in spite of BPPV being a common problem, it took patients many weeks to reach the final diagnosis. The study highlighted the importance of early diagnosis through increased awareness among the clinicians dealing with the concussion, as early diagnosis and treatment will provide quick recovery in a large number of patients [8].

Children who present with head injury often end up having a brain CT scan due to concern from ED physicians about the possibility of heaving intracranial bleeding or fracture. Both of the patients in this study underwent a brain CT scan, which was reported as normal. By appropriate training about the management of BPPV, exposure to radiation can be prevented [9].

Despite being a common medical condition, patients typically suffer for months before receiving appropriate management. This delay may be due to a deficiency in clinicians’ knowledge and skills [6]. The American Academy of Otolaryngology-Head and Neck Surgery has endorsed physicians’ training in diagnosing and treating BPPV through physical maneuvers [9]. In a study conducted among emergency physicians about the treatment of BPPV, ED physicians were asked about their current practice of managing patients with BPPV. Of the physicians, 85% stated that they treated with medications only, 11% offered physical maneuvers along with medication, and only 4% recommended physical maneuvers as the primary treatment of BPPV [10,11].

Postgraduate medical training in the management of BPPV is mostly limited to specific specialties that regularly manage vestibular problems, such as ENT. In contrast, clinicians who typically manage BPPV patients are pediatricians, ED physicians, and family physicians who may have little or no experience with BPPV, which may lead to delays in diagnosis and treatment and may prolong concussion recovery [4].

It is important that clinicians who routinely care for children with head injuries, such as pediatricians, ED physicians, and family physicians, are trained in the art of managing BPPV so that early diagnosis and treatment are offered to prevent unnecessary investigation and long-term disability. The training can be offered through blended learning, using Gagne’s nine steps of instructional design and other methods [12,13].

## Conclusions

Concussion following head injury in rugby and other contact sports is common. It can lead to BPPV, which can cause severe disabling symptoms of recurrent vertigo and vomiting associated with certain head movements. Posterior canal BPPV is the most common subtype. BPPV can be easily diagnosed with the Dix-Hallpike maneuver and treated with the Epley or other bedside maneuvers. The American Academy of Neurology does not recommend the use of imaging or medication for the diagnosis and treatment of BPPV. Increased awareness among sports physicians can expedite diagnosis and treatment and prevent long-term disability.
